# 

**Published:** 1890-02

**Authors:** 


					﻿. Vol. X.
FEBRUARY, 1890.
NO. 27
THE
pOMEOPATHlC pHY^lClAp,
A Monthly Journal of Homoeopathic Materia Medica
and Clinical Medicine.
“ He is a freeman whom.trtUh makes free, and all are slaves beside.”
“Seek the Truth: come whence it may, cost what it will."
EDITED AND PUBLISHED BY
WALTER: ML JAMESy ML Eh?
AND
George h. Clark, m. d.
ASSISTED BY THE FOLLOWING CONTRIBUTORS:
E. T. Balch, M.D., South Bend, Wash.
Clarence Willard Butler, M. D..
Montclair, N. J. .	[York.
Prof Edmund Carleton, M. D_, New
Daniel W. Clausen, M. D., Philada.
Stuart Close, M. D., Brooklyn.
Edward Cranch, M. D., Erie, Pa.
W. S. Gee, M. D.. Hyde Park, Ill. ■
. Harlyn Hitchcock, M. D., New York.
Clarence C. Howard,M;D., New York.
Samuel A. Kimball, M. D., Boston.
Edmund J. Lee, M. D., Former Editor,
Philada.
A. McNeil, M. D., San Francisco.
C. F. Nichols. M. D., Boston.
Mahlon Preston, M. D., Norristown.
i George W. Sherbino, M. D., Abilene,
Texas.
I C. Carleton Smith, M. D., Philada.
1 Wm Steinkauf, M. D., St. Charles, Mo,.
PHILADELPHIA :
No. 1125 SPRUCE STREET.
LONDON-
ALFRED HEATH & CO., Sole Agents for Great Britain,
114 Ebury Street, Eaton Square.
Yearly Subscription, $2.60, invariably in advance. Single numbers, 26 cts.
Entered at the Philadelphia Post-Office at Second-Class Mail Matter.
				

